# Effect of Red and Blue Light on Anthocyanin Accumulation and Differential Gene Expression in Strawberry (*Fragaria* × *ananassa*)

**DOI:** 10.3390/molecules23040820

**Published:** 2018-03-30

**Authors:** Yunting Zhang, Leiyu Jiang, Yali Li, Qing Chen, Yuntian Ye, Yong Zhang, Ya Luo, Bo Sun, Xiaorong Wang, Haoru Tang

**Affiliations:** 1 College of Horticulture, Sichuan Agricultural University, Chengdu 611130, China; asyunting@gmail.com (Y.Z.); jianglysicau@gmail.com (L.J.); lyl016107@126.com (Y.L.); supnovel@gmail.com (Q.C.); yeyuntian@sicau.edu.cn (Y.Y.); zhyong@sicau.edu.cn (Y.Z.); luoya945@163.com (Y.L.); sunadam011@163.com (B.S.); 2 Institute of Pomology and Olericulture, Sichuan Agricultural University, Chengdu 611130, China

**Keywords:** light quality, anthocyanins, proanthocyanidins, gene expression, strawberry

## Abstract

Light conditions can cause quantitative and qualitative changes in anthocyanin. However, little is known about the underlying mechanism of light quality-regulated anthocyanin accumulation in fruits. In this study, light-emitting diodes (LEDs) were applied to explore the effect of red and blue light on strawberry coloration. The results showed contents of total anthocyanins (TA), pelargonidin 3-glucoside (Pg3G) and pelargonidin 3-malonylglucoside (Pg3MG) significantly increased after blue and red light treatment. Pg3G was the major anthocyanin component in strawberry fruits, accounting for more than 80% of TA, whereas Pg3MG accounted for a smaller proportion. Comparative transcriptome analysis was conducted using libraries from the treated strawberries. A total of 1402, 5034, and 3764 differentially-expressed genes (DEGs) were identified in three pairwise comparisons (red light versus white light, RL-VS-WL; blue light versus white light, BL-VS-WL; blue light versus red light, BL-VS-RL), respectively. Photoreceptors and light transduction components remained dynamic to up-regulate the expression of regulatory factors and structural genes related to anthocyanin biosynthesis under red and white light, whereas most genes had low expression levels that were not consistent with the highest total anthocyanin content under blue light. Therefore, the results indicated that light was an essential environmental factor for anthocyanin biosynthesis before the anthocyanin concentration reached saturation in strawberry fruits, and blue light could quickly stimulate the accumulation of anthocyanin in the fruit. In addition, red light might contribute to the synthesis of proanthocyanidins by inducing *LAR* and *ANR*.

## 1. Introduction

Anthocyanins, water-soluble pigment compounds belonging to the flavonoid class, are widely distributed in plants, which facilitate pollination, seed dispersal and stress resistance. They are also important contributors to the organoleptic qualities of colored fruits, vegetables, and flowers. Additinoally, anthocyanins have shown multiple biological activities and exhibit antioxidant, anticarcinogenic, antimicrobial, and antiviral properties [1,2]. For these reasons, interest among plant breeders, the food industry, and consumers in developing anthocyanin-rich foods has intensified.

Anthocyanins are structurally composed of an anthocyanidin aglycone bound to one or more sugar moieties. There are about 17 anthocyanidins found in nature, whereas only six of them, namely, pelargonidin (Pg), cyanidin (Cy), delphinidin (Dp), peonidin (Pn), petunidin (Pt), and malvidin (Mv), occur most frequently in plants [3,4]. Anthocyanins are the end-products of a distinct branch that exists in the late stage of the flavonoid biosynthetic pathway, mainly involved in DFR (dihydroflavonol 4-reductase), leucoanthocyanidin oxidase (LDOX), ANS (anthocyanidin synthase), and UFGT (UDP-glucose: flavonoid 3-*O*-glucosyltransferase) biosynthetic enzymes [5]. It has been reported that most of the anthocyanin pathway enzymes are encoded by small gene families, and usually, one or two members of each gene play a crucial role [6]. The expression of these structural genes relies on the transcriptional activity of MBW ternary complex, which consists of R2R3-MYB and bHLH (MYC-like basic helix–loop–helix) transcription factors, together with WD40-repeat proteins. In vegetative tissues, anthocyanin biosynthesis could be modulated by different MBW complexes involving PRODUCTION OF ANTHOCYANIN PIGMENT1 (PAP1)/MYB75 and PAP2/MYB90, in combination with TRANSPARENT TESTA8 (TT8)/bHLH042, ENHANCER OF GLABRA3 (EGL3)/bHLH002, GLABRA3 (GL3)/bHLH001, and TRANSPARENT TESTA GLABRA1 (TTG1)/WD40 protein [7,8]. R2R3-MYBs are considered as key regulators of anthocyanin biosynthesis and have been well characterized in model plants and fruit trees, such as Arabidopsis [9], petunia [10], strawberry [11], apple [12], grape [13], and lychee [14]. MYB R3 repeat (with a conserved motif (D/E)LX2(R-K)X3LX6LX3R) is necessary for interaction with the N-terminal MYB-interacting region (MIR) of the bHLH. Both MYBs and bHLH have the capability of DNA binding, but the target gene specificity of the MBW complex seems to be more conferred by the MYB protein, while WD40 proteins rather seem to be a docking platform, as they can interact with interact with diverse proteins [15]. The WRKY transcription factors (TTG2/PH3) have been shown to cooperate with the MBW complex, highlighting the importance of transcriptional regulation in the control of flavonoid biosynthesis [16,17]. More recently, WRKY41 was demonstrated to repress anthocyanin accumulation by controlling the expression of regulatory and structural genes involved in anthocyanin biosynthesis in *Arabidopsis thaliana* and *Brassica napus* [18].

Light is one of the most important environmental factors for plant growth and development. Plants rely on it as their source of energy and have evolved mechanisms to perceive light signals, including light intensity, specific wavelengths, photoperiod, and direction. Shading and bagging experiments in various fruit species, such as lychee [19], grapevine [20], apple [21], and pear [22], have demonstrated that light exposure is required to stimulate anthocyanin accumulation and fruit coloration through up-regulation of anthocyanin biosynthetic genes. It has been generally documented that a longer photoperiod can increase the content of anthocyanins and other flavonoids [23,24]; however, inconsistent results have also been reported in some plants. The contents of quercetin and kaempferol were higher at 12 h day lengths under different temperature compared to that at 24 h day lengths [25]. Higher plants can sense specific light wavelengths (also known as light quality or light spectrum) spanning from ultraviolet-B (UV-B) to far-red wavelengths. Arrays of photoreceptors, including UV RESISTANCE LOCUS 8 (UVR8) induced by UV-B, cryptochromes (CRY1, CRY2, CRY3), and phototropins (PHOT1, PHOT2) absorbing ultraviolet-A (UV-A)/blue light, and phytochromes (PHYA-E) responding to red/far-red light, have been reviewed [26]. Photoreceptors regulate photomorphogenic development by suppressing the RING-finger type ubiquitin E3 ligase CONSTITUTIVE PHOTOMORPHOGENIC 1 (COP1) activity, which is directly associated with SUPPRESSOR OF PHYA (SPA) and other COP1 cofactors [27]. ELONGATED HYPOCOTYL 5 (HY5) is a direct target of COP1 and has been linked to the activation of transcription factors and structural genes in the flavonoid pathway in response to light [28,29,30]. In addition, some PHYTOCHROME INTERACTING FACTORs (PIFs) family members were found to be able to interact with phytochromes and participate in anthocyanin biosynthesis by binding the promoters of related genes [31,32].

Strawberry (*Fragaria* × *ananassa*) is popular due to its unique flavor, attractive appearance, and nutritional value, and known as a functional dietary food rich in folate, vitamin C, anthocyanins and other bioactive compounds. Particularly, anthocyanins are natural colorants with health-promoting properties, which have received much attention. Light is one of the most important factors that affects anthocyanin accumulation. However, uneven coloration and defective quality as a result of inadequate anthocyanins often happen in strawberry cultivation with poor light conditions. Thus, supplemental light is necessary. Red and blue light have been generally considered to have the strongest effects on plant growth and metabolism. In this paper, we explored the effect of blue and red light on the anthocyanin accumulation and differentially expressed genes in strawberry, expecting to have a better understanding of the role of light quality in anthocyanin biosynthesis and provide scientific and theoretical basis for strawberry cultivation.

## 2. Results

### 2.1. Anthocyanin Accumulation in Response to Different Light Quality

**Figure 1:**
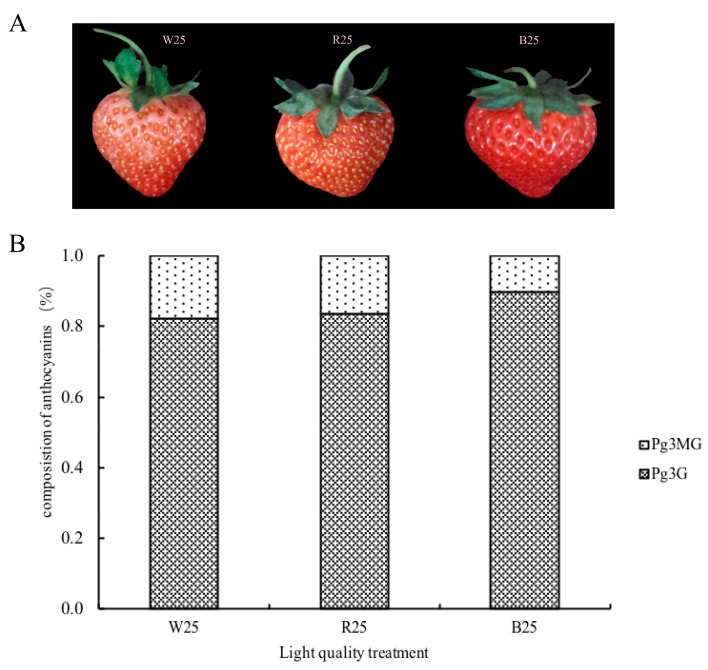
Strawberry phenotypes (**A**) and the composition of anthocyanins (**B**) after white, red, and blue light treatment. W25, R25 and B25 indicate that fruit samples treated by white, red, and blue light were collected on the 25th day after flowering. Pg3G, Pelargonidin 3-glucoside; Pg3MG, pelargonidin 3-malonylglucoside.

Pelargonidin 3-glucoside (Pg3G), pelargonidin 3-malonylglucoside (Pg3MG) and total anthocyanins (TA) content in strawberry fruit after white light (WL), red light (RL), and blue light (BL) treatment were obtained (Table S1). Compared to the control (WL), RL and BL significantly induced anthocyanin accumulation (Figure 1A, Table S1). The highest contents of TA (136 µg·g^−1^) and Pg3G (122.18 µg·g^−1^) were detected in fruit treated with BL (Table S1). Under all light conditions, Pg3G was the major anthocyanin and accounted for more than 80% of TA, whereas Pg3MG made up a smaller percentage. RL and BL can increase the Pg3MG content, but the proportion of Pg3MG in total anthocyanins decreased in both treatments (Figure 1B, Table S1).

### 2.2. Transcriptome Sequencing and Gene Mapping

To obtain a comprehensive overview of the *Fragaria* × *ananassa* fruit transcriptomic responses to light quality, six cDNA libraries, two replicates for each treatment (W251, W252, R251, R252, B251, B252), were constructed. In Table 1, we summarized the evaluation of the sequencing data. Over 46 million clean reads were generated after removing adapters, ambiguous nucleotides, and low-quality reads. More than 91.84% of the reads had a quality score over Q30 (error rate < 0.001). The mean CG content in the transcripts was approximately 50%. Additionally, 47.57–49.01% of the clean reads were mapped into the reference sequences derived from the *Fragaria vesca* genome. The reference genome used was the diploid, while our strawberry samples were the octoploid. The potential differences existed between the reads in the octoploid and the reference diploid, which led to a low mapping rate.

### 2.3. Global Analysis of Differential Gene Expression and Function Annotation

**Figure 2:**
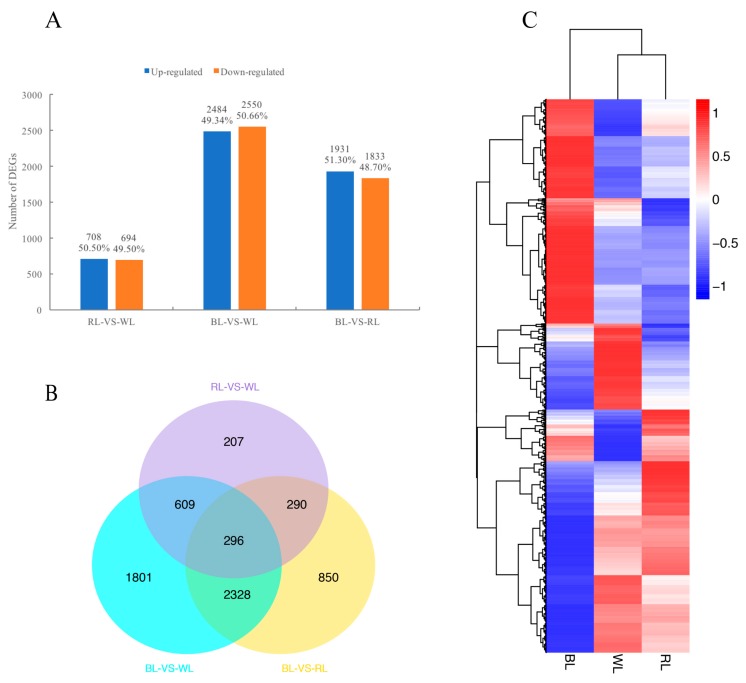
Differential gene expression in response to light quality. (**A**) Numbers of DEGs in pairwise comparisons of the three libraries; (**B**) Venn diagram showing DEG distributions; and (**C**) expression profile clustering. The color scale at the right represents re-processed log10 (FPKM+1) using Pheatmap, representing the relative expression level. The expression variance for each gene is indicated by different colors ranging from low (blue) to high (red). WL, RL, and BL indicates samples treated by white light, red light, and blue light, respectively.

After the gene expression levels were normalized by FPKM, the Pearson correlation coefficient was calculated to ensure the reliability of expression data. This analysis showed that all R^2^ values between biological replicates were larger than those outside the biological replicates (Figure S1). Differentially-expressed genes (DEGs) were analyzed by the DESeq R package (v.1.10.1), based on a negative binomial distribution (*p*_adj_ < 0.05). Through pairwise comparisons (the second treatment listed in the pairwise comparison as the reference), a total of 1402 DEGs were identified between RL and WL, including 708 (50.50%) up-regulated genes and 694 (49.50%) down-regulated genes. Among 5034 DEGs of BL and WL, there were 2484 (49.34%) up-regulated genes and 2550 (50.66%) down-regulated genes. Between BL and RL conditions, 3764 DEGs were detected; of these, 1931 (51.30%) were up-regulated, and 1833 (48.70%) were down-regulated (Figure 2A). As seen in the Venn diagrams showing the relationships of DEGs among the different comparisons (Figure 2B), 850, 1801, and 207 genes were specifically expressed in BL-VS-RL, BL-VS-WL, and RL-VS-WL, respectively, whereas 296 DEGs were shared by all three groups. Subsequently, DEGs from the three treatments were processed by hierarchical clustering. The result showed samples treated by WL and RL were clustered together, indicating their gene expression similarities (Figure 2C).

Gene Ontology (GO) enrichment analysis was performed to further identify the function of the DEGs in the pairwise comparisons (RL-VS-WL, BL-VS-WL, and BL-VS-RL). The genes were sorted into three main GO categories for molecular function, biological process, and cellular component. In RL and WL, most of the differentially-expressed genes that were either up-regulated or down-regulated were significantly classified to some specific Go terms. The top significantly enriched GO subcategories in the biological process category were “metabolic process”, and “single-organism process”. In the molecular function category, “catalytic activity”, “oxidoreductase activity”, and “cofactor binding” were significantly enriched (Figure S2A). Up-regulated DEGs in BL-VS-WL were found mainly in “nitrogen compound metabolic process”, “biosynthetic process”, and “organic substance biosynthesis” within biological processes and cellular components, whereas down-regulated DEGs were mainly enriched in “metabolic process” and “single-organism metabolic process” within biological processes, and “oxidoreductase activity” within the molecular function category (Figure S2B). In BL-VS-RL, only three subcategories (metabolic process, photosystem II and protein phosphorylation) within biological processes and protein kinase activity within the molecular function category were significantly assigned to suppressed genes. In contrast, induced genes were distributed among multiple subcategories in molecular function, biological processes. and cellular components (Figure S2C).

**Figure 3:**
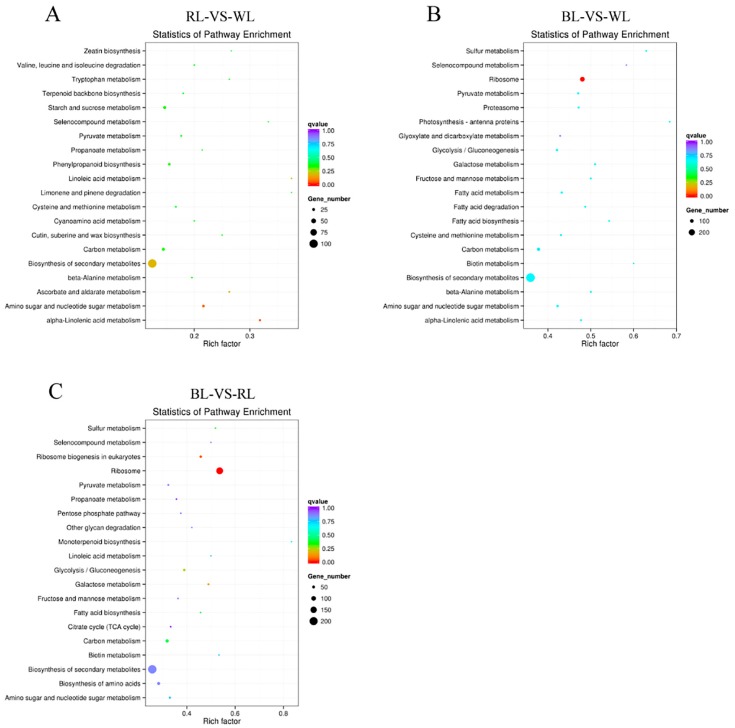
Top 20 enriched KEGG pathways among the annotated DEGs in three pairwise comparisons. (**A**) RL-VS-WL; (**B**) BL-VS-WL; and (**C**) BL-VS-RL. The Y-axis on the left represents KEGG pathways, and the X-axis indicates the enrichment factor. Low q-values are shown in red, and high q-values are depicted in blue. Pathways with q-values less than 0.05 are significantly enriched. The size of the spot reflects the number of DEGs, and the color of the spot corresponds to different q-value ranges. WL, RL, and BL indicates samples treated by white light, red light, and blue light, respectively.

KEGG (Kyoto Encyclopedia of Genes and Genomes, http://www.genome.jp/kegg/) provided a network diagram that could help us to understand the specific roles of DEGs in cell metabolic pathways. In the present study, we searched DEGs in the KEGG pathway database to reveal the top 20 enriched pathways (Figure 3). The following KEGG pathways were enriched in RL-VS-WL: 14 DEGs from “alpha-linolenic acid metabolism” were significantly enriched; 21 DEGs assigned to “amino sugar and nucleotide sugar metabolism”, 102 DEGs related to “biosynthesis of secondary metabolites”, and 10 DEGs allocated to “ascorbate and aldarate metabolism” (Figure 3A) were overrepresented in the dataset. In BL-VS-WL, the most enriched pathway terms were “ribosome”, “sulfur metabolism”, “photosynthesis-antenna proteins”, and “biosynthesis secondary metabolites”, which included 147 DEGs, 17 DEGs, 13 DEGs, and 295 DEGs, respectively (Figure 3B). Notably, genes related to “ribosome” were also enriched in BL-VS-RL (Figure 3C). In addition, flavonoid biosynthesis involved in the coloration of fruits was a common pathway among three pairwise comparisons. Two, eight, and 12 DEGs in RL-VS-WL, BL-VS-RL, and BL-VS-WL, respectively, were assigned to this pathway with 34 genes in the background (Table S2).

### 2.4. Light Signal Perception and Transduction Components

**Figure 4:**
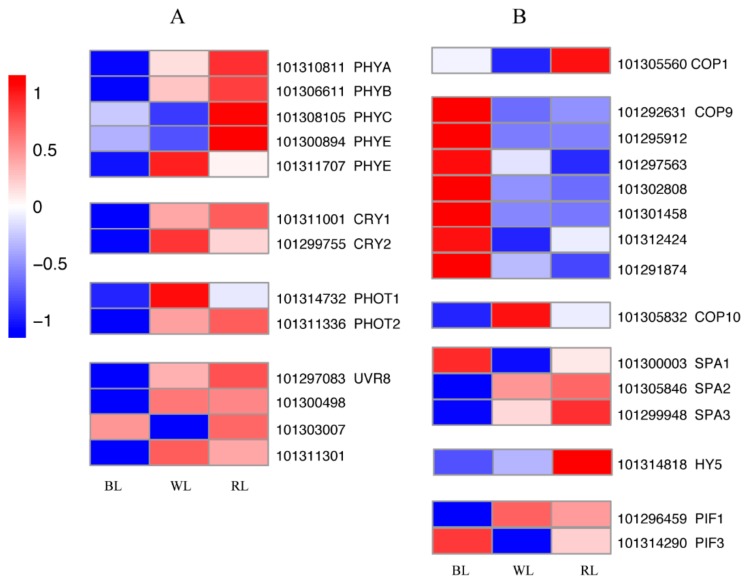
Heat map of gene expression involved in light signal perception (**A**) and transduction (**B**). The color scale at the left represents re-processed log10 (FPKM+1) using Pheatmap, representing the relative expression level. The expression variance for each gene is indicated by colors ranging from low (blue) to high (red). WL, RL, and BL indicates samples treated by white light, red light, and blue light, respectively.

Four different classes of photoreceptors, including phytochromes (PHYs), cryptochromes (CRYs), phototropins (PHOTs), and UV resistance locus 8 (UVR8), have been reported to respond to light quality. We found that the expression levels of all these photoreceptors were down-regulated when the strawberry plants were grown in blue light compared with the other treatment groups (Figure 4A). *COP1*, *COP9*, *COP10*, and *SPAs*, light signal transduction inhibitors, showed different induction levels under WL, RL, and BL. *COP1*, *COP9*, and *COP10* each showed the highest expression in RL, BL and WL, respectively. In the SPA family, the expression level of *SPA1* increased most significantly in fruit treated with BL, whereas *SPA2* and *SPA3* were induced by RL. *HY5*, the positive regulator of the light signaling pathway, exhibited an expression pattern similar to that of *SPA2* and *SPA3*. Fruit exposure to BL and RL resulted in increased *PIF1* transcript levels compared to WL, but *PIF3* expression was inhibited (Figure 4B).

### 2.5. Transcription Factors (TFs)

**Figure 5:**
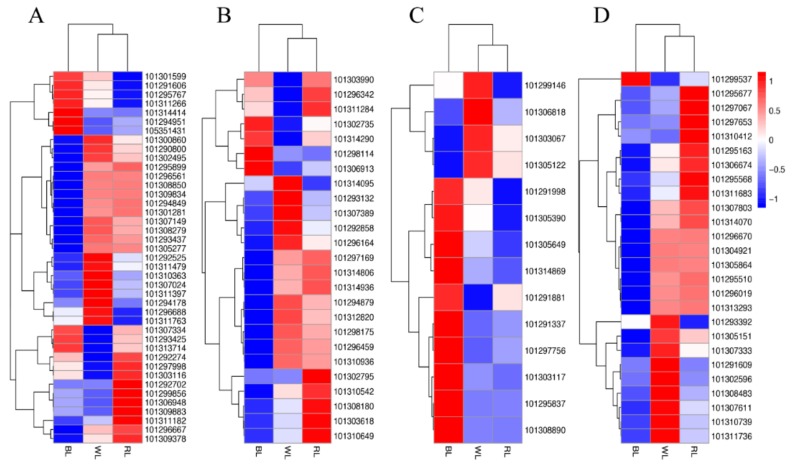
Expression profile clustering of transcription factors related to anthocyanin biosynthesis. (**A**) MYB; (**B**) bHLH, basic helix-loop-helix; (**C**) WD40; and (**D**) WRKY. The color scale at the right represents the re-processed log10 (FPKM+1) using Pheatmap, representing the relative expression level. The expression variance for each gene is indicated by colors ranging from low (blue) to high (red). WL, RL, and BL indicates samples treated by white light, red light, and blue light, respectively.

It has been reported that an MBW complex consisting of MYB, bHLH and WD40 is a critical factor for anthocyanin biosynthesis. In our study, 41 *MYB*, 25 *bHLH*, and 14 *WD40* were identified, respectively (Figure 5A–C). Based on their expression patterns, hierarchical clustering analysis was performed. The results suggested that the RL and WL groups resembled each other. Most of the *MYBs* and *bHLH* genes (containing positive and negative regulatory TFs), except *WD40*, were down-regulated after BL treatment. WRKY transcription factors have also been demonstrated to be implicated in anthocyanin biosynthesis. Regardless of the highest anthocyanin content, almost all *WRKY* expression levels were down-regulated in strawberries exposed to blue light (Figure 5D). Here, those genes definitely related to anthocyanin accumulation in strawberry were included, such as the positive regulatory TF, *MYB10* (Gene ID: 101307024), and the negative regulatory TF, *MYB1* (Gene ID: 101311479).

### 2.6. Structural Genes Involved in the Anthocyanin Pathway

**Figure 6:**
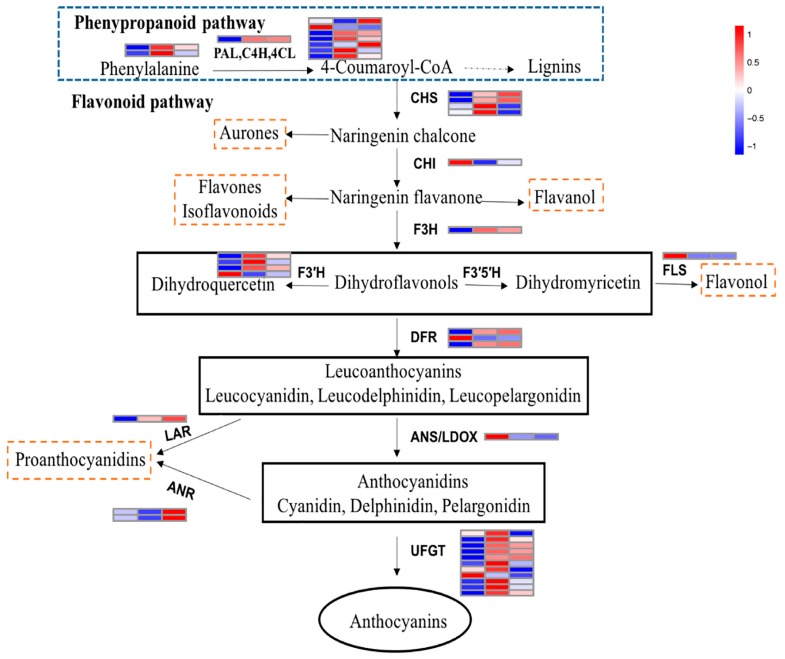
A schematic representation of the flavonoid biosynthetic pathway leading to anthocyanins. The expression pattern of each structural gene involved in anthocyanin synthesis in BL, WL, and RL is arranged from left to right. WL, RL, and BL indicates samples treated by white light, red light, and blue light, respectively. The color scale represents re-processed log10 (FPKM+1) using Pheatmap, representing the relative expression level. The expression variance for each gene is indicated by colors ranging from low (blue) to high (red).

The flavonoid biosynthetic pathway that proceeds via the phenylpropanoid pathway leading to anthocyanins is well characterized. We investigated the expression levels of 39 transcripts related to the 13 genes encoding enzymes involved in this pathway (Figure 6). *PAL*, *4CL*, *CHS*, *F3′H*, *DFR*, *ANR*, and *UFGT* have multiple gene family members. The results showed that BL and RL affected the expression of almost all genes (*PAL*, *C4H*, and *4CL*) that are responsible for precursor biosynthesis. Of early biosynthetic genes in anthocyanin synthesis, two members of *CHS* genes were induced by RL, contrasting with their having the lowest expression level in BL. However, the highest expression level of *CHI* was detected under BL. The expression levels of almost all *F3H* and *F3′H* transcripts were suppressed in BL and RL treatment. Strong signals for transcripts of the late biosynthetic genes *DFR* and *ANS* were clearly seen under BL, which was consistent with the high content of anthocyanins in plants under this treatment. The transcript abundance of structural genes in specific branches of the Proanthocyanidins (PA) and flavonol pathways were also measured. The RL contributed more to PA accumulation (Figure S3) via high expression of *LAR* and *ANR*, whereas BL significantly increased *FLS* transcript abundance, which is related to flavonol synthesis.

### 2.7. Validation of Digital Expression Profiles by qRT-PCR

To further confirm the reliability and accuracy of Illumina RNA-Seq analysis results, we randomly selected 11 identified DEGs involved in light signal perception and transduction, as well as the transcription factor and structure genes related to the flavonoid biosynthesis pathway, and assessed their expression patterns by qRT-PCR. As shown in Figure S4, all selected genes displayed expression profiles similar to those observed in RNA-Seq data. Moreover, qRT-PCR and RNA-Seq were positively correlated. Therefore, all the above evidence suggested that the RNA-Seq data were reliable.

## 3. Discussion

The genetic background of the plant is the main determinant of anthocyanin biosynthesis, whereas the external environment, including biotic and abiotic factors, can cause quantitative and qualitative changes in the composition of these compounds [33,34]. The light quality plays an important role for anthocyanin accumulation, independent of photoperiod and light intensity [26]. LEDs are characterized by a narrow light-emitting spectrum, enabling the focus of the light exposed to plants using only the required wavelength [35]. This method is more efficient in converting electric power to light power, and it also enables the irradiation of plants at a close distance for a long time because LEDs are cold light illuminators [36]. Currently, blue and red LEDs in biologically-active visible light regions have been widely introduced to regulate vegetable and fruit quality [37,38,39], but they have been less applied to fruit in vivo. Strawberry plants have a small size, and they do not require a very high light intensity (100–300 µmol·m^−2^·s^−1^) [40]. Therefore, it is possible and valuable to investigate the influence of light quality on strawberry fruit coloration using LEDs with single-peak blue and red light in artificial climate chambers where other environmental factors can be controlled.

In our study, the results showed that blue and red light treatment resulted in significantly higher levels of total anthocyanin (TA), and the highest content was achieved in blue light-treated fruit (‘Toyonaka’), followed by red light treatment, which was consistent with responses observed in grape and bilberry fruit [41,42]. Differences in the anthocyanin profile (consisting of Pg3G and Pg3MG) were detected in each treated group. In general, the main anthocyanin in strawberry (‘Toyonaka’) was Pg3G, like other cultivars [43,44], accounting for 89.84%, 83.60%, and 82.41% in BL, RL, and WL treatments, respectively. This indicated that increased total anthocyanins in strawberry fruits in response to light quality mainly depended on Pg3G production; the secondary anthocyanin (Pg3MG) showed a distinct increase at a quantitative level under RL and BL treatment, but accounted for a smaller proportion of total anthocyanins than in plants under the control treatment (WL). Researchers have found that light quality affects anthocyanin biosynthesis differently [45]. It is generally documented that short wavelengths in the range of blue and UV light seem to be the prominent wavelengths impacting the increase in anthocyanin content [26,37,46]. Nevertheless, evidence suggesting that light with long wavelengths can increase the intensity of the red coloration also exists [47,48,49]. In strawberry, blue light can induce different cultivar anthocyanin accumulation [50,51], whereas red and yellow selective plastic films can increase the temperature and play a positive role in anthocyanin biosynthesis [49]. Therefore, optimization of anthocyanin concentration using light quality should take species, cultivars, and microenvironment variation into consideration.

Plants utilize multiple photoreceptors to perceive different wavelengths, which then trigger a series of signal transduction events, and finally lead to physiological responses [19,52,53]. In the present study, transcriptome analysis was conducted to further understand the relationship between strawberry pigmentation and monochromatic visible light (red and blue) at the transcriptional level. We found that four types of photoreceptors, including cryptochromes (*CRY1*, *CRY2*, and *CRY3*) and phototropins (*PHOT1* and *PHOT2*), were down-regulated in fruit treated with blue light. Kadomura-Ishikawa (2013) demonstrated that expression patterns of photoreceptor genes decrease gradually during fruit development from the small green to red stages in strawberry, with the exception of *PHOT2* [50], so the reduction in photoreceptor transcription levels could be considered a sign of full fruit coloration. Consistently, anthocyanin biosynthesis is typically associated with blue light through CRYs [54,55]. However, the regulation of anthocyanin synthesis via CRYs requires the activity of PHYs for full expression [56,57,58]. CRYs also need interplay with UVR8 to mediate target gene expression under natural environmental conditions [59,60]. Thus, other types of light apart from blue light could also significantly affect anthocyanin synthesis in plants. In this study, red light induced the anthocyanin accumulation and expression levels of *PHYs* and other photoreceptors. Moreover, the expression level of *HY5*, a positive photomorphogenic regulator, was also significantly up-regulated by red light.

In light-inducible anthocyanin biosynthesis, MYBs seem to play a pivotal role in structural gene expression and anthocyanin accumulation [61,62]. *Lilium regale LrMYB15* transcription ceased completely when plants were kept in shaded conditions, and the colors of the flower buds faded, indicating that transcription of this gene is under the control of light [61]. Potato *StMYBA1* requires light to activate anthocyanin biosynthesis in transgenic tobacco [62]. These light-sensitive MYBs have also been identified in many fruits, such as grape, apple, pear, lyhee, and strawberry [14,63,64,65,66], and are usually activated by HY5 [28,29,67]. In strawberry, *FaMYB10* has a role as a signal transduction mediator from light perception to anthocyanin synthesis [66], and it was also identified in our DEGs (Gene ID: 101307024). Most of the MYBs involved in anthocyanin biosynthesis interact with bHLHs and WD40s to form the MBW regulatory complex. However, the role of bHLH and WD40 partners in light-regulated anthocyanin biosynthesis is not yet clear. In addition, some experiments have noted that WRKYs are also involved in the regulation of this pathway [68,69]. We showed that *MYB*, *bHLH*, and *WRKY* expression patterns were similar to photoreceptors under different light quality treatments. Noticeably, most of these regulators had a low transcription abundance in fruit treated with blue light, which resulted in a decrease in the expression level of numerous structural genes. However, the highest anthocyanin content was detected in blue light treatment. Possibly, these genes decreased at this stage that anthocyanin concentration reached saturation, suggesting that blue light could promote the rapid accumulation of anthocyanin in strawberry fruit. In red and white light treatment, light signal perception and transduction components were dynamic to keep the regulatory factors and structural genes of anthocyanin biosynthesis at a high expression level. Moreover, red light not only elevated the anthocyanin contents, it might also contribute to the synthesis of proanthocyanidins by inducing the *LAR* and *ANR*. Hence, these results also indicated that the light is an essential environmental factor for anthocyanin biosynthesis before anthocyanin concentration reaches a plateau in fruit.

## 4. Materials and Methods

### 4.1. Plant Materials

‘Toyonaka’ (*Fragaria* × *ananassa*) seedlings were grown in 15 cm × 13 cm pots filled with a 2:2:1 (*v*/*v*/*v*) mixture of nutrient soil, garden soil, and perlite and subjected to routine management from September 2014 in the greenhouse of Sichuan Agricultural University. Subsequently, on the seventh day after flowering, potted seedlings of uniform size were randomly divided into three groups, transferred into assigned growth chambers and maintained on a photoperiod of 8 h dark at 16 °C and 16 h light at 25 °C at 75% relative humidity. White (control), red (730 nm), and blue (450 nm) light-emitting diodes (LEDs) were placed at the top of the chambers to irradiate strawberry seedlings at 100 μmol·m^−2^·s^−1^ until fruits were harvested. Fruit samples from three biological replicates (at least three fruits per replicate) for each treatment were collected on the 25th day after flowering and then stored at −80 °C after immediately freezing in liquid nitrogen.

### 4.2. Anthocyanin and Proanthocyanidin Determination

Approximately 1.0 g of frozen samples was ground to a fine powder in liquid nitrogen, then soaked in 5 mL of methanol containing 1% (*v*/*v*) hydrochloric acid overnight at 4 °C. After centrifugation at 5000× *g* for 20 min, the supernatant was transferred into a brown volumetric flask. Then, the residue was re-homogenized twice using extraction buffer. Finally, the supernatants were combined and brought to a final volume of 10 mL. One milliliter of the extract was passed through a 0.45 μm membrane filter before HPLC analysis. Determination of anthocyanins was performed on an Agilent 1260 HPLC system (Agilent Technologies, Palo Alto, CA, USA) equipped with variable wavelength detectors (VWD) and a ZORBAX SB-C18 column (150 mm × 4.6 mm, 5 μm). Mobile phases consisting of ultra-pure water (A), acetonitrile (B), and formic acid (C) were used to carry out a linear gradient elution. The chromatographic conditions were as follows: 0 min, 100% A; 13 min, 78% A + 20% B + 2% C; 20 min, 58% A + 40% B + 2% C; and 25 min, 100% A. The flow rate was 1 mL·min^−1^; the injection volume was 10 μL; and the C18 column was maintained at 30 °C. Anthocyanins were detected at 520 nm by VWD.

Proanthocyanidin content was determined using an improved DMAC (4-dimethylaminocinnamaldehyde) method that has been described previously [70]. A 0.5–1.0 g sample of strawberry powder was added into 20 mL extraction solution, which was a mixture of acetone, deionized water, and acetic acid (150:49:1 *v*/*v*/*v*), placed on a shaker for 1 h and subsequently centrifuged at 10,000× *g* for 20 min at 12 °C. The supernatant was collected for analysis at 640 nm in a 96-well microplate reader (Thermo Fisher Scientific, Waltham, MA, USA).

Standards were purchased from Sigma (St. Louis, MO, USA), and all samples were prepared in triplicate.

### 4.3. RNA Extraction, cDNA Library Preparation and Illumina Sequencing

Total RNA was isolated from each sample using the improved CTAB (cetyltrimethylammonium bromide) method [71]. The quality and quantity of the total RNA were analyzed by electrophoresis on a 1% agarose gel and using a NanoPhotometer^®^ spectrophotometer (Implen, Westlake Village, CA, USA) and the RNA Nano 6000 Assay Kit on a Bioanalyzer 2100 system (Agilent Technologies, Palo Alto, CA, USA).

cDNA libraries were obtained from white, red, and blue light-treated strawberries in two biological replicates (W251, W252, R251, R252, B251, B252). A total amount of 3 µg RNA per sample was used as input material. Sequencing libraries were generated using NEBNext^®^ Ultra™ RNA Library Prep Kit for Illumina^®^ (NEB, Ipswich, MA, USA). Briefly, mRNA was purified from total RNA by Oligo(dT)-attached magnetic beads and sheared randomly using fragmentation buffer. Subsequently, the fragment templates were used to synthesize first strand cDNA by adding random hexamer primers and M-MuLV Reverse Transcriptase (RNase H^−^) followed by the generation of the second-strand cDNA using dNTPs, buffer, DNA polymerase I, and RNase H. Then, the double-stranded cDNA was purified with AMPure XP beads and sequentially modified by end-repairing, poly-A tail-adding and Illumina adapter-ligating before selecting for fragment size with AMPure XP beads. Finally, the cDNA libraries were PCR-amplified and assessed using a Qubit 2.0 Flurometer (Life Technologies, Carlsbad, CA, USA), a Bioanalyzer system (Agilent Technologies, Palo Alto, CA, USA) and Q-PCR methods. Transcriptome sequencing was performed on the Illumina HiSeq 2500 platform (Illumina, San Diego, CA, USA), and 125/150 bp paired-end raw reads were generated.

### 4.4. RNA-Seq Data Preprocessing Reads Mapping

Raw data (raw reads) in fastq format were first processed with in-house Perl scripts. In this step, clean data (clean reads) were obtained by removing reads containing adapter, reads containing poly-N (ratio of “N” accounted for more than 10%) and low-quality reads (bases of Qphred ≤ 20, Q20, which accounted for more than 50%) from the raw data. At the same time, the Q20, Q30, and GC contents of the clean data were calculated. All downstream analyses were based on clean data with high quality. Reference-based strategy to assemble a transcriptome is the most straightforward method, which requires a high-quality reference genome. However, the whole-genome sequence of *Fragaria* × *ananassa* has not been published. *Fragaria vesca* is the most plausible progenitor of *Fragaria* × *ananassa* and the availability of its genome offers the possibility of mapping the transcriptomic data of *Fragaria* × *ananassa* to a reference genome, which contributes greatly to advances in molecular genetic analysis and identification of genes related to agriculturally-important traits in *Fragaria* × *ananassa* [72,73]. Hence, clean reads were mapped to the *Fragaria vesca* reference genome (https://www.rosaceae.org/organism/Fragaria/vesca) [74]. Reference genome and gene model annotation files were downloaded directly from the genome website. An index of the reference genome was built using Bowtie v2.2.3, and paired-end clean reads were aligned to the reference genome using TopHat v2.0.12 with the default constraint of two mismatches.

### 4.5. Differentially-Expressed Gene (DEG) Identification and Functional Annotation

RNA-Seq gene expression levels were normalized by calculating FPKM (fragments per kilobase of transcript sequence per million base pairs sequenced) [75]. Differential expression analysis was performed using the DESeq R package v1.10.1 after the Pearson correlation between samples was analyzed [76]. DESeq provides statistical routines for determining differential expression in digital gene expression data using a model based on the negative binomial distribution. The resulting *p*-values were adjusted using Benjamini and Hochberg’s approach (1995) applied in multiple hypothesis testing for controlling the false discovery rate (FDR) [77]. Genes with an adjusted *p* < 0.05 found by DESeq were assigned as differentially expressed. Then, DEGs were subjected to Gene Ontology (GO) and Kyoto Encyclopedia of Genes and Genomes (KEGG) enrichment analysis implemented by the Goseq R package and KOBAS software, respectively, for functional annotation, and corrected *p*-values less than 0.05 were considered significantly enriched. Heatmaps were generated using the among-gene normalized FPKM values as an indicator of gene expression values. Before drawing the diagram, average FPKM was obtained between two replicates, and the log 10 transformed (FPKM+1) were used for drawing the map with the Pheatmap R package, and the relative expression level was represented by colors ranging from low (blue) to high (red) for each gene across the entire dataset.

### 4.6. Quantitative Real-Time PCR (qRT-PCR) Analysis

qRT-PCR was used to validate the gene expression patterns obtained by RNA-Seq on the CFX96 real-time PCR system (Bio-Rad, Hercules, CA, USA). cDNA for each sample was generated using the PrimeScript™ RT reagent Kit with gDNA Eraser (Takara Shuzo, Kyoto, Japan), according to the manufacturer’s instructions. Gene-specific primers used for verification of DEGs were listed in Table S3. All PCR reactions contained 0.4 μL of each primer (0.4 μM), 5 μL SYBR Premix (Takara Shuzo, Kyoto, Japan), 1 μL cDNA template, and 3.2 μL of RNase-free water in a final volume of 10 μL. The reaction protocol was set with three-step cycling conditions: 95 °C for 3 min, followed by 40 cycles of 95 °C for 10 s, 60 °C for 30 s and 72 °C for 15 s. A melting curve analysis was inserted after the final cycle, ramping from 65 °C to 95 °C (increment 0.5 °C/5 s). Relative fold changes in gene expression were calculated by the 2^−ΔΔCT^ method, and the *FaActin* gene (GenBank: LC017712.1) was selected as the internal control to normalize the raw data.

## 5. Conclusions

In summary, both blue and red light were effective in promoting anthocyanin accumulation in strawberry fruit. Additionally, red light might contribute to the synthesis of proanthocyanidins by inducing *LAR* and *ANR*. We analyzed the global transcriptome modification of these fruits and, thus, provided an overview of the effect of light quality during strawberry coloration. The identification and functional analysis of a large number of DEGs suggested that light quality as a signal could simultaneously stimulate various metabolic networks, including the anthocyanin biosynthesis pathway, coordinated by transcriptional factors.

## Figures and Tables

**Table 1:** Summary statistics of the sequencing data.

Sample Name	Raw Reads	Clean Reads	Clean Bases	Error Rate (%)	Q20 (%)	Q30 (%)	GC Content (%)	Total Mapped (%)
W251	51,106,150	49,645,282	7.45 G	0.02	96.75	92.19	46.8	48.01
W252	49,154,200	47,925,342	7.19 G	0.01	96.85	92.33	47.32	48.49
B251	56,427,402	54,927,816	8.24 G	0.02	96.7	92.09	46.89	47.57
B252	48,846,890	47,634,610	7.15 G	0.02	96.57	91.86	46.61	47.80
R251	48,997,928	47,876,282	7.18 G	0.02	96.57	91.84	46.92	47.96
R252	47,859,022	46,555,194	6.98 G	0.01	96.84	92.38	47.18	49.01

Q20, Q30: The percentage of bases with a Phred value of >20 or 30, Phred = −10log_10_(e); W251, W252; R251, R252; B251, B252 represent two biological replicates for each treatment, respectively.

## Supplementary Materials

The following are available online. Figure S1: Pearson correlation between samples. W251, W252; R251, R252; B251 and B252 represents two biological replicates for each treatment, respectively, Figure S2: The most enriched GO terms of up- and down-regulated genes in RL-VS-WL (**A**), BL-VS-WL (**B**), and BL-VS-RL. Asterisks indicate the significance of over-presentation (corrected *p* < 0.05), Figure S3: The content of proanthocyanidins in strawberry fruits after different light quality treatments. Each value represents the mean ± standard error. Different lower-case letters indicate significant differences based on one-way analysis of variance in SPSS 23.0 followed by the Duncan test (*p* < 0.05). WL, RL, and BL indicate samples treated by white light, red light, and blue light, respectively, Figure S4: Expression analysis of 11 differentially-expressed genes related to anthocyanin biosynthesis in strawberry fruit treated with different light quality. The left y-axis shows the relative gene expression levels analyzed by qRT-PCR and the right y-axis indicates the corresponding RNA-Seq expression data. Each value in the histogram represents the mean ± standard error. The scatter plot of 11 selected genes based on the log2 of the gene expression ratios from qRT-PCR and RNA-seq results indicates the correlation between them. WL, RL, and BL indicates samples treated by white light, red light, and blue light, respectively, Table S1: Content and proportion of anthocyanins in strawberry fruits after different light treatments, Table S2: DEGs enriched in flavonoid biosynthesis pathway in three pair comparison, Table S3: Primers used in RT-qPCR.
