# Supplementary material for: Effect of Red and Blue Light on Anthocyanin Accumulation and Differential Gene Expression in Strawberry (Fragaria × ananassa)
Source: Molecules. 2018 Mar 30;23(4):820. doi: 10.3390/molecules23040820 (PMC6017741; doi:10.3390/molecules23040820)
Supplement: Supplementary file 1 [file molecules-23-00820-s001.zip › Supplementary materials-proofreading/Supplementary information.docx]

Effect of Red and Blue Light on Anthocyanin Accumulation and Differential Gene Expression in Strawberry (*Fragaria* × *ananassa*)

Yunting Zhang ^1,†^, Leiyu Jiang ^1,†^, Yali Li ^1^, Qing Chen ^1^, Yuntian Ye ^1^, Yong Zhang ^1^, Ya Luo ^1^,
Bo Sun ^1^, Xiaorong Wang ^1,2,^* and Haoru Tang ^1,^*

^1^ College of Horticulture, Sichuan Agricultural University, Chengdu 611130, China;
[asyunting@gmail.com](mailto:asyunting@gmail.com) (Yu.Z.); [jianglysicau@gmail.com](mailto:jianglysicau@gmail.com) (L.J.); [lyl016107@126.com](mailto:lyl016107@126.com) (Y.Li.); [supnovel@gmail.com](mailto:supnovel@gmail.com) (Q.C.); [yeyuntian@sicau.edu.cn](mailto:yeyuntian@sicau.edu.cn) (Y.Y.); [zhyong@sicau.edu.cn](mailto:zhyong@sicau.edu.cn) (Yo.Z.); [luoya945@163.com](mailto:luoya945@163.com) (Y.Lu.); [sunadam011@163.com](mailto:sunadam011@163.com) (B.S.)

^2^ Institute of Pomology and Olericulture, Sichuan Agricultural University, Chengdu 611130, China

***** Correspondence: Wangxr@sicau.edu.cn (X.W.); htang@sicau.edu.cn (H.T.); Tel: +86-028-8629-1949 (H.T.)

**^†^** These authors contributed equally to this work.

**Supplementary Figures**

**
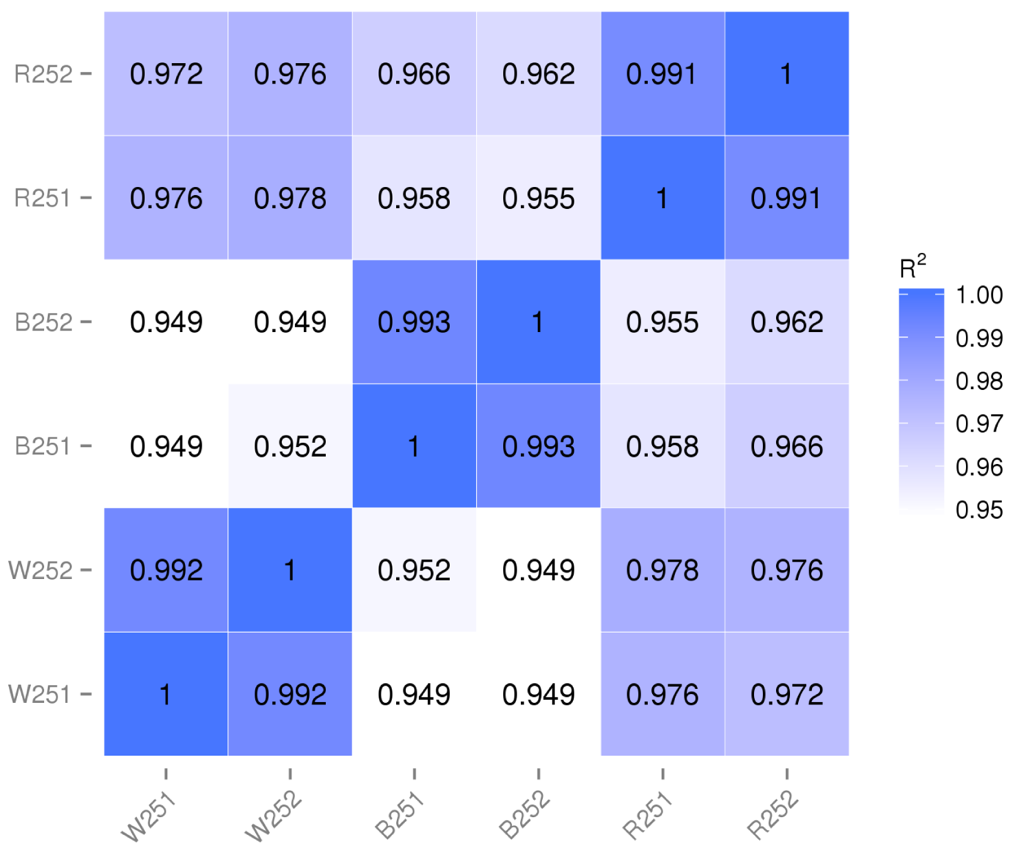
**

**Figure S1. Pearson correlation between samples.** W251, W252; R251, R252; B251, B252 represents two biological replicates for each treatment, respectively.


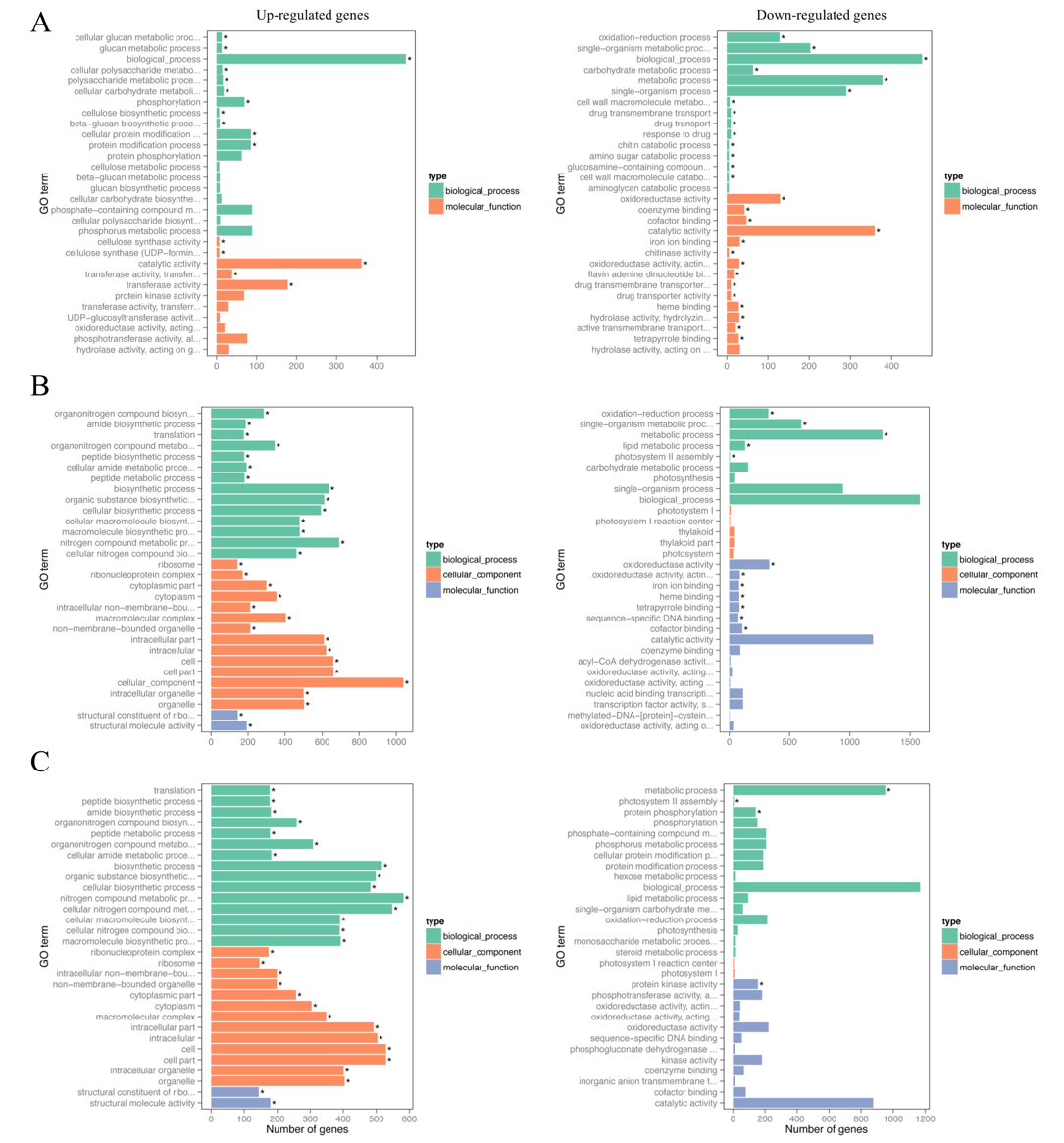


**Figure S2. The most enriched GO terms of up- and down-regulated genes in RL-VS-WL（A），BL-VS-WL（B），and BL-VS-RL.** Asterisk indicates the significance of over-presentation (corrected p-value < 0.05).

**Figure S3. The content of proanthocyanidins in strawberry fruits after different light quality treatment.** Each value represents the mean ± standard error. Different lower-case letters indicate signiﬁcant differences based on one-way analysis of variance in SPSS 23.0 followed by the Duncan test (P < 0.05). WL, RL and BL indicates samples treated by white light, red light and blue light, respectively.


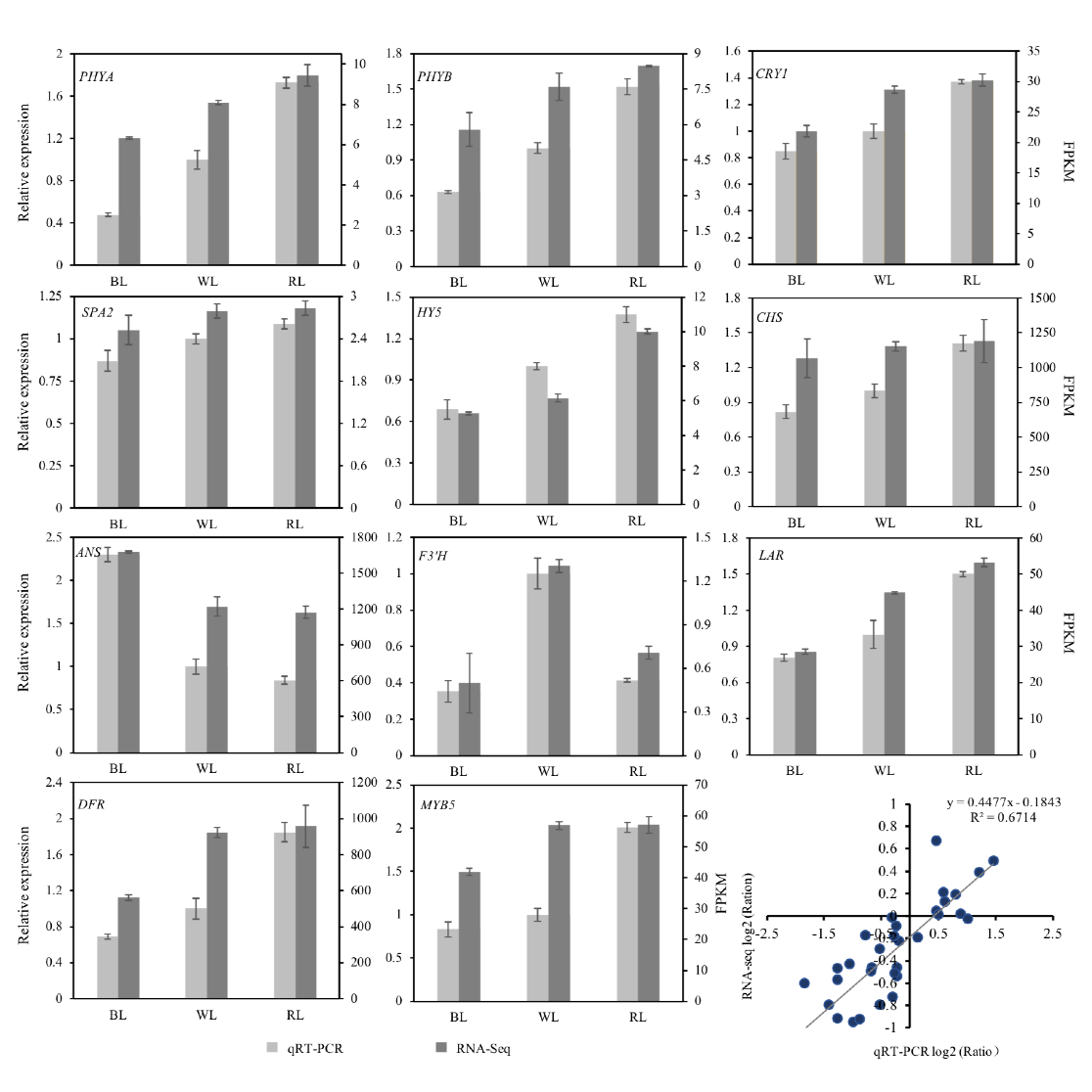


Figure S4. Expression analysis of 11 differentially expressed genes related to anthocyanin biosynthesis in strawberry fruit treated with different light quality. The left y-axis shows the relative gene expression levels analyzed by qRT-PCR and the right y-axis indicates the corresponding RNA-Seq expression data. Each value in the histogram represents the mean ± standard error. The scatter plot of 11 selected genes based on the log2 of the gene expression ratios from qRT-PCR and RNA-seq results indicates the correlation between them. WL, RL and BL indicates samples treated by white light, red light and blue light, respectively.

**Supplementary Tables**

**Table S1. Content and proportion of anthocyanins in strawberry fruits after different light treatments.**

| Treatment | TA | Pg3G | |  | Pg3MG | |
| --- | --- | --- | --- | --- | --- | --- |
|  | Content (µg·g-1) | Content (µg·g-1) | Percent (100%) |  | Content (µg·g-1) | Percent (100%) |
| W25 | 54.97±0.43 c | 45.30±0.4 c | 82.41 |  | 9.67±0.18 c | 17.59 |
| R25 | 117.09±1.60 b | 97.89±1.32 b | 83.60 |  | 19.20±0.31 a | 16.40 |
| B25 | 136.00±1.44 a | 122.18±1.10 a | 89.84 |  | 13.82±0.38 b | 10.16 |

TA, total anthocyanins; Pg3G, pelargonidin 3- glucoside; Pg3MG, pelargonidin 3-malonylglucoside. W25, R25 and B25 indicates that fruit samples treated by white, red and blue light were collected on the 25^th^ day after flowering. Each value represents the mean ± standard error. Different lower-case letters within a column indicate signiﬁcant differences based on one-way analysis of variance in SPSS 23.0 followed by the Duncan test (P < 0.05).

**Table S2. DEGs enriched in flavonoid biosynthesis pathway in three pair comparison.**

| Group | Sample number | Background number | P-value | Corrected P-value | UniGenes | KO | Entrez ID |
| --- | --- | --- | --- | --- | --- | --- | --- |
| RL-VS-WL | 2 | 34 | 0.833699042545 | 1.0 | 101295967 101298438 | fve:101295967 fve:101298438 | 101295967 101298438 |
| BL-VS-WL | 12 | 34 | 0.428141091536 | 0.999998628408 | 101292744 101294342 101298456 101297735 101310387 101308284 101293749 101293459 101295967 101303591 101300182 101306809 | fve:101292744 fve:101294342 fve:101298456 fve:101297735 fve:101310387 fve:101308284 fve:101293749 fve:101293459 fve:101295967 fve:101303591 fve:101300182 fve:101306809 | 101292744 101294342 101298456 101297735 101310387 101308284 101293749 101293459 101295967 101303591 101300182 101306809 |
| BL-VS-RL | 8 | 34 | 0.564464389854 | 0.99960947464 | 101292744 101294342 101297735 101310387 101308284 101293749 101306809 101298438 | fve:101292744 fve:101294342 fve:101297735 fve:101310387 fve:101308284 fve:101293459 fve:101306809 fve:101298438 | 101292744 101294342 101297735 101310387 101308284 101293749 101306809 101298438 |

**Table S3. Primers used in RT-qPCR.**

| Gene name | Gene_id | Forward primer (5´ to 3´) | Reverse primer (5´ to 3´) | Product size (bp) |
| --- | --- | --- | --- | --- |
| *PHYA* | 101310811 | CTTTGAGGAGTCGGGCAGTT | CGGGACAGCATGACTAACCA | 241 |
| *PHYB* | 101306611 | CACACCTACTGAAGCGCAGA | TATAAGCAGCAGCCATCCCG | 155 |
| *CRY1* | 101311001 | ATGCTGTTCAATCCCGCAGA | CATCCACAAGGGCTCTCCAG | 99 |
| *SPA2* | 101305846 | GGAGTGTTTTGTGAACCCGC | CTCGTGAACTCCCACCACTC | 80 |
| *HY5* | 101314818 | CAAGACCAAGCCACGAGC | TCCCTGCCTGAGACCGATG | 173 |
| *CHS* | 101298162 | TGGTGCCGCAGCCATAATTGTTG | GCCCAGGAACATCTTTGAGGAG | 170 |
| *ANS* | 101308284 | GTGAGGGAGAAATGTAGGGAGGAT | GGAGATGCCGTGGTTGATAAGG | 81 |
| *F3’H* | 101296377 | AAAACACTCCAACCTAACCCACAC | CAAAACCAGGGCGACGAGAA | 184 |
| *DFR* | 101293749 | AACGAAGTGATAAAGCCAACA | GTAAACACCAACCTCCGAAC | 89 |
| *MYB5* | 101308850 | ACTCCTCGGCAATAGATGGTC | GATTGAGTGGCTTGTGGGTTCT | 143 |
| *MYB11* | 101296688 | GTCTTTGATAGCGGGTAGGC | CATTTGGTGGTTTGTGGTGAG | 140 |
